# Epigastric port retrieval of the gallbladder following laparoscopic cholecystectomy is associated with the reduced risk of port site infection and port site incisional hernia: An updated meta-analysis of randomized controlled trials^☆^

**DOI:** 10.1016/j.amsu.2020.05.017

**Published:** 2020-05-25

**Authors:** Sumit Sood, Anja Imsirovic, Parv Sains, Krishna K Singh, Muhammad S Sajid

**Affiliations:** a Department of General Surgery, University Hospitals of Coventry and Warwickshire, United Kingdom; b Department of Digestive Diseases & Gastrointestinal Surgery, Brighton & Sussex University Hospitals NHS Trust, The Royal Sussex County Hospital, Eastern Road, Brighton, West Sussex, BN2 5BE, United Kingdom

**Keywords:** Cholecystectomy, Laparoscopic, Cholecystitis, Gallstone disease, Keyhole surgery

## Abstract

**Aims:**

The objective of this article is to compare the surgical outcomes for epigastric port or umbilical port retrieval of the gallbladder (GB) following laparoscopic cholecystectomy (LC).

**Methods:**

The data retrieved from the published randomized, controlled trials (RCT) comparing the surgical outcomes for epigastric port or umbilical port retrieval of the GB following LC was analysed using the principles of meta-analysis. The summated outcome of continuous variables was expressed as standardized mean difference (SMD) and dichotomous data was presented in odds ratio (OR).

**Results:**

Eight RCTs on 2676 patients comparing the surgical outcomes for epigastric port or umbilical port retrieval of the GB following LC were analysed. In the random effects model analysis using the statistical software Review Manager 5.3, the GB retrieval through epigastric port was associated with the reduced duration of operation (SMD, 0.41; 95% CI, 0.18, 0.64; z = 3.52; P = 0.0004). Epigastric retrieval was also associated with reduced risk of surgical site infection (OR, 1.95; 95% CI, 0.75, 5.11; z = 1.36; P = 0.17), and port site incisional hernia (OR, 4.22; 95% CI, 0.43, 41.40; z = 1.24; P = 0.22) compared to umbilical port retrieval though it did not reach statistical significance. The need for port enlargement to retrieve the GB was similar in both groups. In contrast, the umbilical port retrieval of the GB was associated with significantly less post-operative pain (SMD, −0.51; 95% CI, −0.95, −0.06; z = 2.24; P = 0.03), reduced GB perforation rate, reduced port site bleeding rate and reduced difficulty in GB retrieval.

**Conclusion:**

GB retrieval through epigastric port following LC has clinically proven advantage of reduced retrieval site infection rate, lower operation time and incisional hernia rate but at the cost of increased pain at 24 h, higher risk of GB perforation, port site bleeding and technical difficulties.

## Highlights

• An updated meta-analysis providing better evidence on gall bladder extraction port following laparoscopic cholecystectomy(LC).
• Epigastric port retrieval of gallbladder following LC has advantage of reduced rate of both infection and incisional hernia.
• Umbilical port retrieval of gallbladder following LC has advantage of reduced pain, port site bleeding and perforation risk.
• In conclusion from this meta-analysis epigastric port retrieval seems to be more advantageous compared to the umbilical port.

## 1 Introduction

Laparoscopic cholecystectomy (LC) is a preferred method of gallbladder removal for symptomatic gallbladder stones and for other benign conditions. The use of LC in the management of gallbladder disease has shown several advantages over open cholecystectomy such as reduced postoperative pain, reduced risk of surgical site infections, quicker recovery and reduced incidence of incisional hernia [[1], [2], [3], [4]]. Among most pronounced and commonly listed complications, is the development of incisional hernia at the site of epigastric port or umbilical port. Several published studies have reported that the most frequent location of incisional hernia is the umbilical port site with an incidence ranging from 0.18 to 2.8% [5,6]. Furthermore, in patients with comorbidities such as advanced age, diabetes mellitus, and obesity, the incidence can reach up to 22% [5,6]. This has an impact on overall cost of hospitalization-according to NHS reference cost document; non-elective inpatient hospitalization costs around £1603 per day [7]. In case of prolonged stay due to complications of laparoscopic gallbladder removal, one can only multiply given figures on the cost of the LC.

Another common complication is the surgical site infection (SSI) at port site, which may be as high as 5% [8] when umbilical port site has been used for retrieval of the gallbladder versus 1.6% when epigastric port site has been used following laparoscopic cholecystectomy. The possibility of higher incidence of umbilical port site SSI rate may be related to actual trocar size, umbilical pit containing several organisms, and especially if it is associated with other risk factors of diabetes mellitus, advanced age, obesity and enlargement of facial wound. Umbilical pit related higher risks of developing SSI can potentially be reduced by not contaminating the port site further by gallbladder retrieval through this port [8]. As for now, both umbilical port and epigastric port are being used for retrieval of the gallbladder in LC are usually chosen upon surgeon's preference or local institutional guidelines. The objective of this article is to compare the surgical outcomes for epigastric port versus umbilical port retrieval of the gallbladder following LC.

## 2 Methods

The protocol for this systematic review was established prior to initiation of the study according to the reporting methodology conforms to the PRISMA (Preferred Reporting Items for Systematic reviews and Meta-Analyses) guidelines [9].

### 2.1 Suitability criteria, trial selection and search strategy

Randomised, controlled trials (RCT) were included comparing the umbilical port gallbladder retrieval versus epigastric port gallbladder retrieval following LC. No other study design was considered for review or analysis but quasi-RCTs were also considered because of paucity of patients and RCTs. Studies enrolling patients of any age or gender were included and any other exclusion criteria were not applied. The main interventions were conventional 3 port or four port LC for benign gallbladder diseases. The electronic databases of Embase, Medline through PubMed, the Cochrane Central Register of Controlled Trials (CENTRAL, provider Wiley Online Library) and Open Grey were searched from their inception until September 2019. A combination of the following MeSH terms (Medical Subject Headings) were used: “gallstones”, “cholelithiasis”, “acute cholecystitis”, “chronic cholecystitis”, “gallstone disease”, “biliary dyskinesia”, and “gallbladder dysfunction” in conjunction with “laparoscopic cholecystectomy”, “Keyhole surgery”, and “minimal invasive surgery”. No language restrictions were applied during search of all electronic databases. Eligibility assessment was performed independently in an unblended standardised manner by reviewers. Disagreements were resolved by consensus after consultation with the senior clinician involved in the management of gallbladder diseases for last 24 years.

### 2.2 Data collection and management

Two independent reviewers were involved in study selection. Reviewers were blinded to studies selected for inclusion by the other reviewer. Bibliographic references of published RCTs and systematic reviews or meta-analyses were also thoroughly screened. Data was extracted using a standardised data collection form. One reviewer extracted the data and the second and third reviewers cross-checked the extracted data. The most important variables for data collection were, bibliographic data including date of completion/publication; country of origin; publication status of study; source of funding for trial; trial design; care setting; number of participants randomised to each trial arm and number included in final analysis; eligibility criteria and key baseline participant data including category(s) and location(s); details of treatment regimen received by each group; duration of treatment; details of any co‐interventions; primary and secondary outcome(s) (with definitions and, where applicable, time points); outcome data for primary and secondary outcomes (by group); duration of follow‐up; number of withdrawals (by group) due to adverse events; and adverse events. The primary outcome measure was the incidence of SSI and occurrence of port site incisional hernia which may require either medical or surgical treatment. The data related to the primary outcome measure was collected from all possible published resources such as the abstract, main text, tables or graphs.

### 2.3 Statistical synthesis of the collected data

A fixed-effect model was planned to apply for the synthesis of the data in the absence of heterogeneity. The presence of heterogeneity was evaluated by assessing the consistency of study population, intervention, perioperative care characteristics and method of outcome assessment, by inspecting the forest plots, and by computing the chi^2^ as well as I^2^ values. If significant heterogeneity among the included RCTs was identified, the random-effects model analysis was used as recommended by DerSimonian and Laird [10]. Standardised difference in means (SMD) with a 95% confidence intervals (CIs) were calculated to assess the size of the effect. Where means and p-values were given, we estimated the standard error and the standard deviation by calculating the standard error and t-value using the given degrees of freedom. The standard error and standard deviation were obtained from confidence intervals by using the formula suggested by the Cochrane Collaboration [[11], [12], [13], [14], [15], [16]]. Pooled odds ratios (ORs) with 95% CIs were calculated to measure the effect of each type of procedure on dichotomous variables. Publication bias was planned to assess the symmetry of funnel plots if at least 8 trials were included in the meta-analysis. Statistical analysis was performed using RevMan 5.3 (Review Manger 5.3, The Nordic Cochrane Centre, Copenhagen, Denmark). Trial sequential analysis was performed to assess the possibility of type I error and to compute the information size. The Land and DeMets method were used to construct monitoring boundaries and set adjusted thresholds for statistical significance [17].

### 2.4 Methodological assessment

Risk of bias of the included studies was assessed using Cochrane Collaboration's tool [[11], [12], [13], [14], [15], [16]]. This tool considers random sequence generation, allocation concealment, blinding of participants, personnel and outcome assessors; incomplete outcome data, selective outcome reporting and other potential threats to validity.

## 3 Results

### 3.1 Literature search outcome

**Fig. 1:**
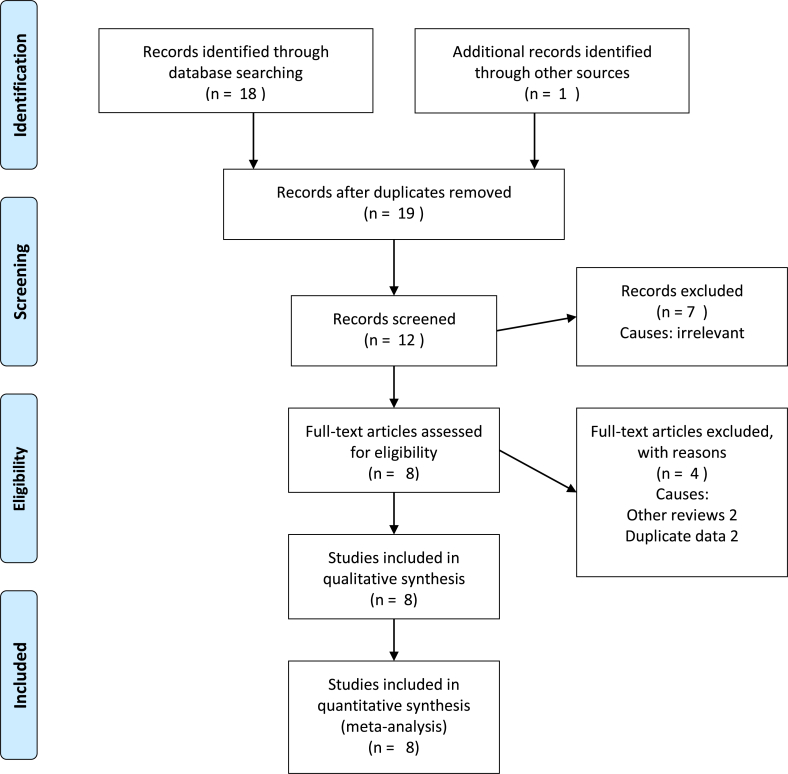
PRISMA flow chart.

**Fig. 2:**
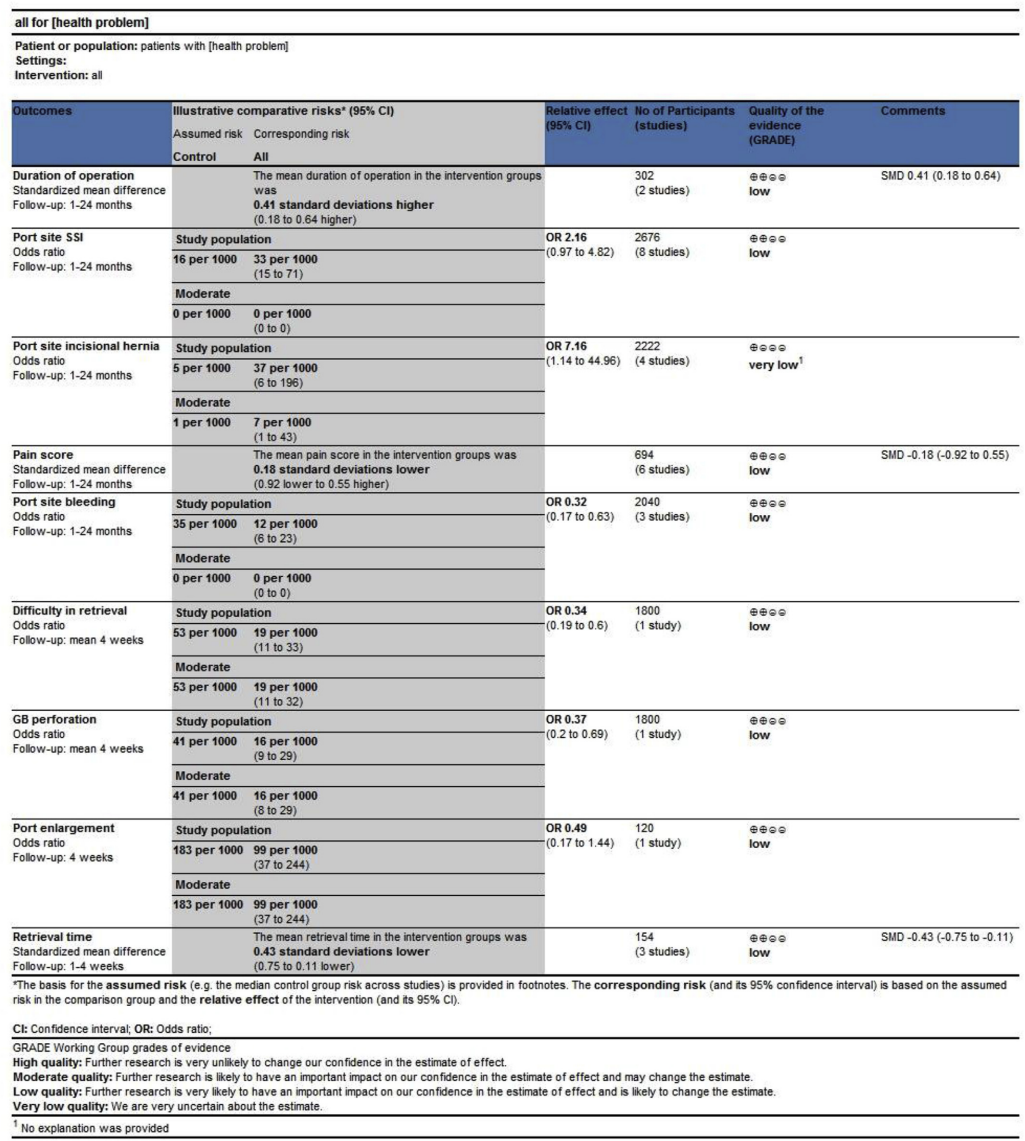
GradePro summary of evidence.

The PRISMA flow chart to explain the literature search strategy and trial selection is given in Fig. 1. Eight RCTs [[18], [19], [20], [21], [22], [23], [24], [25]] on 2676 patients undergoing LC were retrieved from the search of standard medical electronic databases. The quality of the reported and included trials was inadequate due to the lack of using optimum randomization technique, blinding approach, power calculations and intention-to-treat analysis. The generated evidence on the background of these methodologically flawed trials may be considered biased Table 2 and of low quality, but the best available baseline evidence concurrently Fig. 2. The characteristics of the included RCTs are given in Table 1.

**Table 1:** Characteristics of included trials.

Trial	Year	Country	Patients	Age in years	Female: Male	Follow up time	Trial running time
Ahmad et al.^18^ UPR EPR	2014	Pakistan	60	48.5 ± 7.4 48 ± 5.7	21:9 15:15	24 h	2013–2014
Bashir et al.^19^ UPR EPR	2014	Pakistan	94	47.94 ± 7.394 46.84 ± 5.640	33:17 19:21	24 h	2013–2014
Hajong et al.^20^ UPR EPR	2017	India	100	33.48 ± 10.6 31.10 ± 7.8	34:16 35:15	24 h	2016–2017
Kaya et al.^21^ UPR EPR	2017	Italy	120	51 ± 13.2 49 ± 15.4	39:21 42:18	30 days	2016
Li et al.^22^ UPR EPR	2018	China	182	62.1 ± 17.1 61.5 ± 15.9	56:25 55:27	24 months	2014–2017
Memon et al.^23^ UPR EPR	2014	Pakistan	1800	45	3:1	Not reported	2012–2014
Shakya et al.^24^ UPR EPR	2015	India	200	38.9 35.7	17:3 13:7	24 h	2015
Siddiqui et al.^25^ UPR EPR	2012	Pakistan	120	40.6 ± 12.6 42.5 ± 10	45:15 47:13	36 h	2010

UPR: Umbilical port retrieval.

EPR: Epigastric port retrieval.

**Table 2:** Trial quality indicators.

Trial	Randomization technique	Power calculations	Blinding	Intention-to-treat analysis	Concealment	Inclusion criteria	Exclusion criteria
Ahmad et al.^18^	By random number generation	Not reported	Not reported	Not reported	Not reported	- Any gender − 16-60 years - Gall stones - Chronic cholecystitis	Immuno-compromised BMI >40 kg/m Gall bladder cancer
Bashir et al.^19^	By random number generation	Not reported	Reported	Not reported	Not reported	All gender Gallstones	Not reported
Hajong et al.^20^	Block- first 50 in Group 1, second 50 in group 2	Not reported	No data	Not reported	Not reported	All gender − 18-80 years - Gall stones GB polyps	Not reported
Kaya et al.^21^	Not reported	Not reported	Not reported	Not reported	Not reported	- All gender − 18-80 years - Gall stones	- Carcinoma of GB - Acute cholecystitis - Pregnancy - BMI >40 - Immunosuppressed
Li et al.^22^	Computer based random generation	Reported	Reported	Reported	Reported	-All gender −18-80 years -Gall stones	- Carcinoma of GB - Acute cholecystitis - Pregnancy - BMI >40 - Immunosuppressed
Memon et al.^23^	Not reported	Not reported	Not reported	Not reported	Not reported	No data	- Children, patients with obstructive jaundice - Carcinoma of GB
Shakya et al.^24^	Random selection in theatre. Quasi- RCT	Not reported	Not reported	Not reported	Not reported	Cholelithiasis	Not reported
Siddiqui et al.^25^	Lottery slips by third person	Yes	Single blinding	Not reported	Sealed envelopes	All gender − 18-75 years - Gall stones	- Acute cholecystitis - Mucocele of GB - Carcinoma of GB - Conversion to open - chronic users of analgesics and steroids

### 3.2 Treatment effect of the intervention

**Fig. 3:**
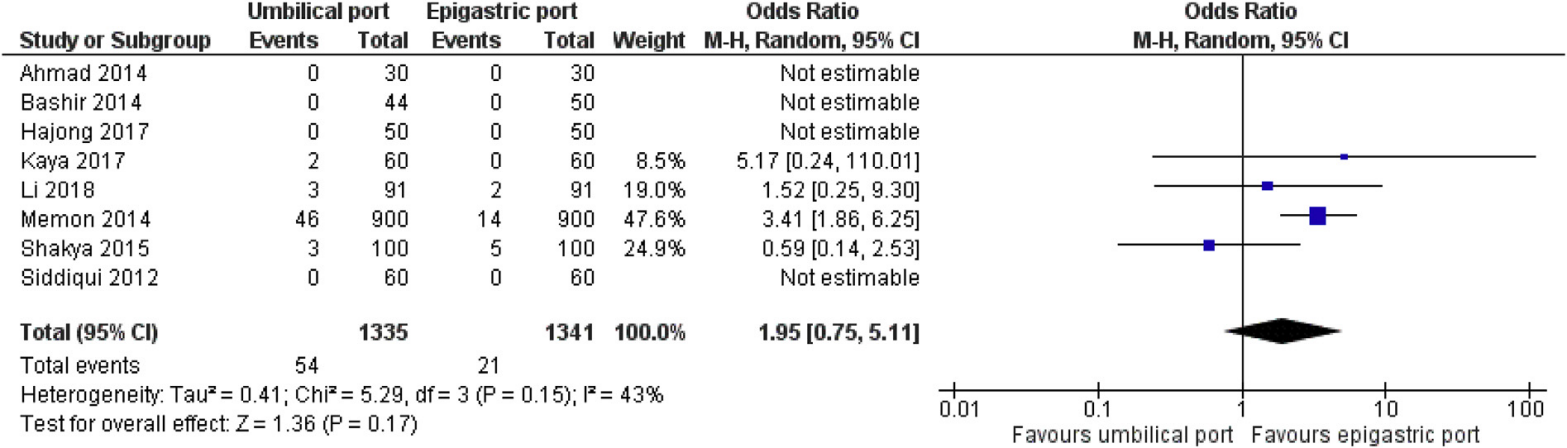
Forest plot for the port site surgical site infection. Odds ratio is shown by 95% confidence interval.

**Fig. 4:**
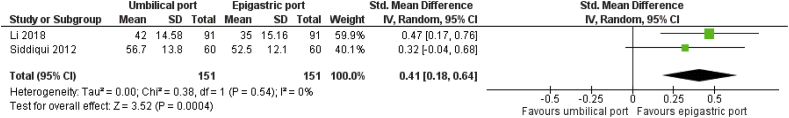
Forest plot for the duration of laparoscopic cholecystectomy. Mean difference is shown by 95% confidence interval.

**Fig. 5:**
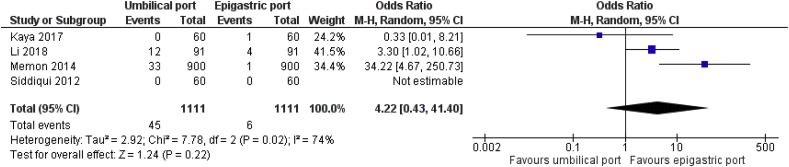
Forest plot for the risk of incisional hernia. Odds ratio is shown by 95% confidence interval.

Eight RCTs on 2676 patients comparing the surgical outcomes for epigastric port or umbilical port retrieval of the gallbladder following LC were analysed. In the random effects model analysis using the statistical software Review Manager 5.3, the gallbladder retrieval through the epigastric port was associated with the reduced duration of operation (SMD, 0.41; 95% CI, 0.18, 0.64; z = 3.52; P = 0.0004; Fig. 4). Epigastric retrieval was also associated with reduced risk of surgical site infection (OR, 1.95; 95% CI, 0.75, 5.11; z = 1.36; P = 0.17; Fig. 3), and lower risk port site incisional hernia (OR, 4.22; 95% CI, 0.43, 41.40; z = 1.24; P = 0.22); Fig. 5) compared to umbilical port retrieval though it did not reach clinical significance. There was significant heterogeneity (chi^2^ = 7.78, df = 2, [p = 0.02]; I^2^ = 74%) among trials.

**Fig. 6:**
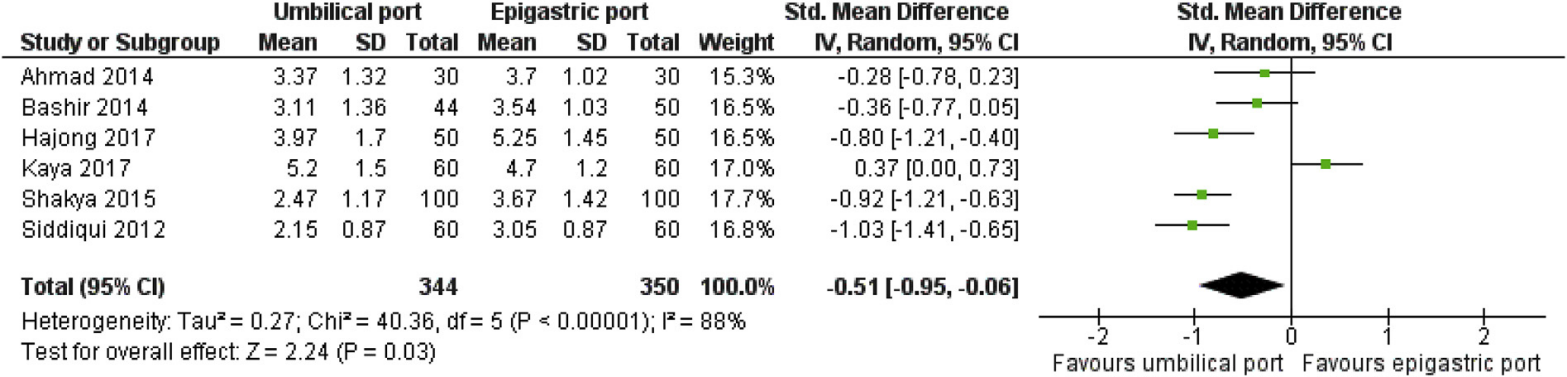
Forest plot for 24 h pain score. Mean difference is shown by 95% confidence interval.

**Fig. 7:**
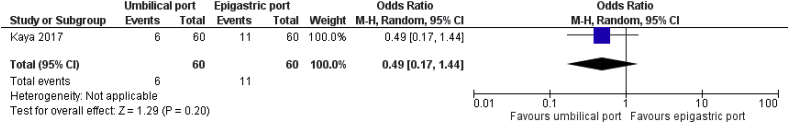
Forest plot for the need of port enlargement for gallbladder retrieval. Odds ratio is shown by 95% confidence interval.

**Fig. 8:**
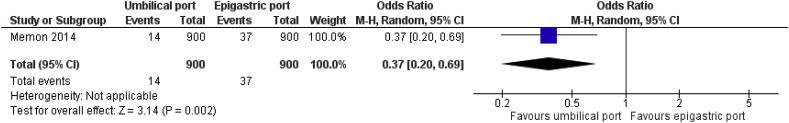
Forest plot for the gallbladder perforation during retrieval. Odds ratio is shown by 95% confidence interval.

**Fig. 9:**
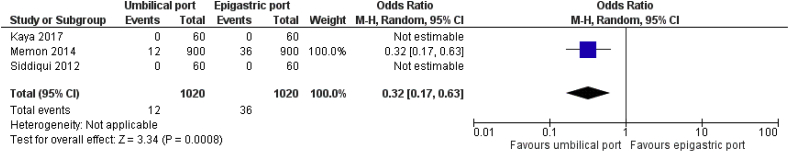
Forest plot for the risk of port site bleeding. Odds ratio is shown by 95% confidence interval.

**Fig. 10:**
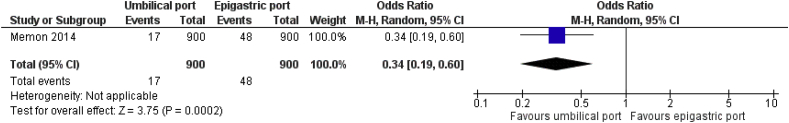
Forest plot for difficulty in retrieval. Odds ratio is shown by 95% confidence interval.

**Fig. 11:**
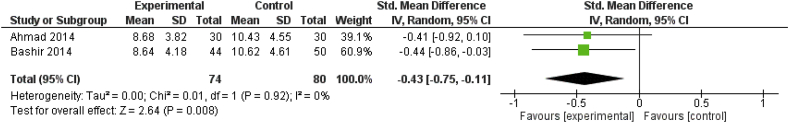
Forest plot for retrieval time. Odds ratio is shown by 95% confidence interval.

The need for port enlargement to retrieve the GB (OR, 0.49; 95% CI, 0.17, 1.44; z = 1.29; P = 0.20; Fig. 7) were similar in both groups. In contrast, the umbilical port retrieval of the GB was associated with significantly less post-operative pain score at 24 h (SMD, −0.51; 95% CI, −0.95, −0.06; z = 2.24; P = 0.03; Fig. 6); reduced GB perforation rate (OR, 0.37; 95% CI, 0.20, 0.69; z = 3.14; P = 0.002; Fig. 8), reduced port site bleeding rate (OR, 0.32; 95% CI, 0.17, 0.63; z = 3.34; P = 0.0008; Fig. 9) and reduced difficulty in GB retrieval (OR, 0.34; 95% CI, 0.19, 0.60; z = 3.75; P = 0.0002; Fig. 10). The umbilical port retrieval takes shorter time compared to epigastric port retrieval (SMD, −0.43; 95% CI, −0.75, −0.11; z = 2.64; P = 0.008; Fig. 11).

## 4 Discussion

### 4.1 Summary of main results

To the best of our knowledge the results of this largest ever meta-analysis of 8 RCTs investigating 2676 patients successfully demonstrate that the retrieval of gallbladder through epigastric port following LC is associated with the reduced risk of port site infection and port site incisional hernia though it failed to reach clinical significance. The duration of operation in case of epigastric retrieval of the gallbladder was also shorter compared to umbilical port retrieval. The need of port enlargement for gallbladder retrieval was statistically similar in both approaches. However, the umbilical port retrieval was technically easy, was less painful at 24 h and was associated with lower risk of gallbladder perforation and lower risk of port site bleeding.

### 4.2 Completeness and application of current study

The findings of current study are resultant from the combined analysis of RCTs of variable quality. There was significant diversity in the inclusion criteria, exclusion criteria and methodological methods reported in included RCTs. Based upon the quality indicators of the Cochrane tool and GradePro, all included trials were of low quality, therefore the results of current study should be read cautiously until a high quality RCTs validate these findings.

### 4.3 Clinical value of current evidence

As shown in Fig. 2 the quality of the evidence is of low and further studies are mandatory before recommending any approach of gallbladder retrieval as a routine technique.

### 4.4 Potential biases in the study

Authors adopted the standard Cochrane Collaboration methodology to perform the statistical analysis, interpretation as well as to present the quality of the resulting evidence. The quality of included RCTs was assessed for risk of bias in case of presence or absence of blinding and at unclear risk of bias in another domain (allocation concealment). The higher risk of bias was mainly attributable to the absence of blinding in all the trials and limited reporting of presence of allocation concealment in included studies. Presence of variable quality of randomization techniques and rare utilization of the power calculations in all included trials provided inadequate strength to generate higher level of evidence. The aforementioned methodological limitations should be also acknowledged before accepting the conclusions of this study.

### 4.5 Comparison with other similar studies

The findings of current meta-analysis of 8 RCTs on 2676 patients undergoing LC are entirely dissimilar to previously published single meta-analysis [26]. Hajibandeh et al. study demonstrated that the gallbladder retrieval via the umbilical port was associated with less postoperative pain and shorter retrieval time. Our study concluded that gall bladder retrieval through epigastric port following LC has clinically proven advantage of reduced retrieval site infection rate, lower operation time and incisional hernia rate but at the cost of increased pain at 24 h, higher risk of GB perforation, port site bleeding and technical difficulties Previously reported the data of only 5 RCTs whereas current study is the largest series of 8 RCTs on 2676 patients. Hajibandeh et al. seem to completely ignore the incidence of port site incisional hernia in the largest prospective cohort study by Memon et al., the reason to which is completely unknown. It reported 0 among 900 cases in Umbilical arm though the original paper quoted 33 in 900, which a very significant finding and more representative of day to day practice. It is acknowledged that this study is prospective cohort study and the included RCT also had limitations as discussed above and hence was included as a quasi-randomised study.

### 4.6 Implications for practice and research

Based upon the findings of current study the gallbladder retrieval following LC through epigastric port has clinically proven advantages of reduced port site infection rate, lower operation time and incisional hernia rate but at the cost of higher risk of pain at 24 h, Gall bladder perforation, port site bleeding and technical difficulties. Because included studies are of low quality and generated evidence may be considered biased. In order to validate current findings; a high quality, high powered, major and multi-centre RCT is mandatory.

## Provenance and peer review

Not commissioned, externally peer reviewed.

## Sources of funding

There was no funding for this paper.

## Ethical approval

Not required.

## Consent

Not applicable.

## Registration of research studies

1. Name of the registry: ResearchRegistry
2. Unique Identifying number or registration ID: reviewregistry889
3. Hyperlink to your specific registration (must be publicly accessible and will be checked). https://www.researchregistry.com/browse-the-registry#registryofsystematicreviewsmeta-analyses/?view_13_page=1&view_13_search=reviewregistry889

## Guarantor

Mr. M S Sajid.

## CRediT authorship contribution statement

**Sumit Sood:** Writing - original draft, Writing - review & editing. **Anja Imsirovic:** Writing - original draft, Writing - review & editing. **Parv Sains:** Writing - original draft, Writing - review & editing. **Krishna K Singh:** Writing - original draft, Writing - review & editing, Formal analysis. **Muhammad S Sajid:** Writing - original draft, Writing - review & editing, Formal analysis.

## Declaration of competing interest

There is no conflict of interest.
