# Supplementary material for: Epigastric port retrieval of the gallbladder following laparoscopic cholecystectomy is associated with the reduced risk of port site infection and port site incisional hernia: An updated meta-analysis of randomized controlled trials
Source: Ann Med Surg (Lond). 2020 May 25;55:244–51. doi: 10.1016/j.amsu.2020.05.017 (PMC7283097; doi:10.1016/j.amsu.2020.05.017)

Figure 1: PRISMA Flow Diagram

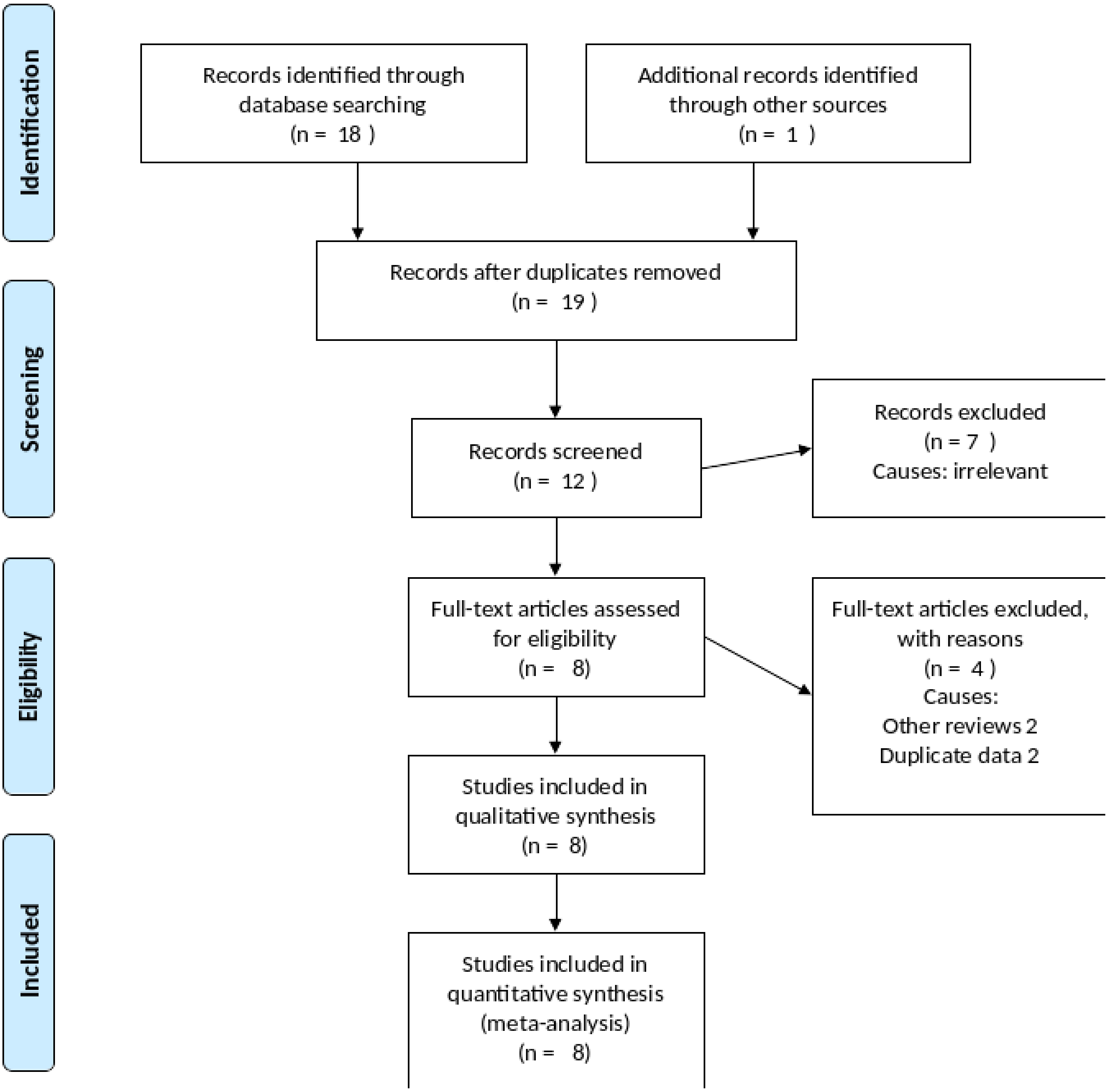

Supplement: Supplementary file 2 [file mmc1.docx]
